# The Landscape of *Salmonella enterica* Serovar Gallinarum–Pullorum Antimicrobial Resistance in Bangladesh's Poultry Industry: A Combined Phenotypic and Molecular Study

**DOI:** 10.1002/mbo3.70328

**Published:** 2026-06-10

**Authors:** Md. Masuk Rahman Kingshuk, Shanzida Binte Alam, Md. Shajedur Rahman, Md. Nurnoby Islam

**Affiliations:** ^1^ Department of Medicine, Surgery, and Obstetrics Hajee Mohammad Danesh Science and Technology University Dinajpur Bangladesh; ^2^ Department of Microbiology and Hygiene Bangladesh Agricultural University Mymensingh Bangladesh

**Keywords:** antimicrobial resistance gene, multidrug‐resistant strain, multiple antibiotic resistance, polymerase chain reaction, *Salmonella enterica* serovar Gallinarum–Pullorum

## Abstract

*Salmonella enterica* serovar Gallinarum–Pullorum is a prevalent poultry‐borne pathogen that could facilitate the zoonotic transmission of multidrug‐resistant (MDR) strains to humans, significantly contributing to the global burden of antimicrobial resistance (AMR). A total of 200 samples were collected from chickens of various flocks suspected of salmonellosis based on clinical diagnosis and isolation via standard bacteriological protocols, of which 138 tested positive for serovar Gallinarum–Pullorum by polymerase chain reaction (PCR). Each PCR‐positive sample's isolates were sent for antimicrobial susceptibility testing, and each PCR‐positive sample's DNA samples were sent for PCR‐based resistance gene detection to evaluate the effectiveness of antimicrobials, MDR, and AMR status. Isolates were sensitive to gentamicin, amoxicillin–clavulanic acid, and cephalexin at rates of 84.78%, 83.33%, and 81.78%, respectively. Conversely, amoxicillin (71.01% resistant) and tetracycline (73.91% resistant) showed the highest rates of resistance. The MDR group accounted for 123 isolates, translating to a commanding combined prevalence of 89.13%, and resistance peaked at four resistant agents, with an incidence of 31.16% (43). Multiple antibiotic resistance (MAR) Index levels varied from 0.1 to 0.7. AML‐SXT‐TE‐NOR and AML‐SXT‐TE‐NOR‐ENR were the most commonly seen patterns, each occurring in five isolates (3.62%). The tetA (74.64%) and the sul1 (73.19%) were the most prevalent resistance genes, with the presence of blaCMY (71.01%), blaTEM (9.42%), blaCTX (7.97%), sul2 (55.80%), dfrA1 (37.68%), aph(3′)‐IIa (42.03%), aac(3)‐VIa (5.80%), aadB (3.62%), floR (35.51%), gyrA (60.87%), and parC (47.83%) genes. This study reveals that *S. enterica* exhibits high levels of AMR, manifesting in MDR phenotypes supported by a robust presence of resistance‐conferring genes.

## Graphical abstract

*Salmonella enterica* serovar Gallinarum–Pullorum isolated from chickens reveals a critical 89.13% prevalence of multidrug resistance. High phenotypic resistance to amoxicillin and tetracycline is genetically underpinned by a robust presence of resistance‐conferring genes (notably *tetA, sul1*, and *blaCMY*), highlighting a significant zoonotic threat to public health.

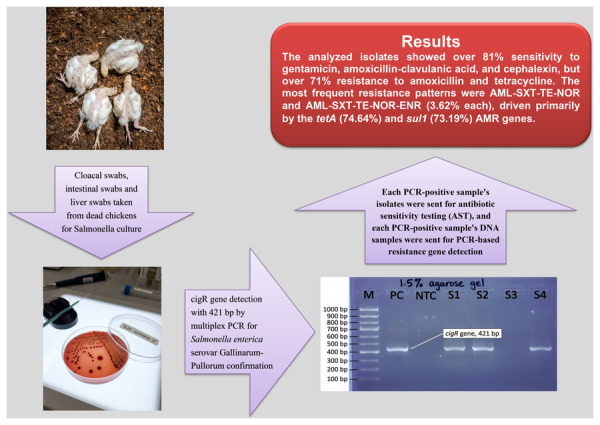

## 1 Introduction

In Bangladesh, as a developing nation, poultry products (meat and eggs) are the primary and most affordable source of animal protein, making them several times less expensive than alternatives. This affordability drives significant demand and has fostered an expansive, highly profitable poultry industry, characterized by a vast number of small‐ to medium‐scale farms operating throughout the country. To ensure eggs and poultry meat remain affordable for consumers, producers face extreme pressure to minimize production costs. In the context of cost minimization, the practice of using antibiotics for both prophylactic (disease prevention, including *Salmonella* spp. control) and therapeutic purposes is often adopted because it is relatively low‐cost compared with alternatives, such as more rigorous biosecurity measures, sanitation programs, or vaccination protocols. However, this extensive and potentially indiscriminate use of antimicrobials in Bangladeshi poultry farms is a critical contributing factor to the selection and development of multidrug‐resistant (MDR) variants of *Salmonella* spp.

Salmonellosis is still one of the most common foodborne zoonoses, posing significant public health concerns worldwide (Shaji et al. 2023). This gut bacterium can spread across all animals, including humans (Tariq et al. 2022), and the ingestion of contaminated chicken meat and eggs is the major source of infection for humans (Alikhan et al. 2022). In the poultry industry, systemic infections and large financial losses are caused by *Salmonella* Gallinarium and *Salmonella* Pullorum, the two most virulent serovars in avian species, and they can spread both vertically from parent flocks to offspring and horizontally from contaminated settings to birds (Tariq et al. 2022). The spread of this disease is facilitated by contemporary husbandry techniques, the industry's regional concentration, high stocking densities, and continuous feeding (Wang et al. 2023; Sanni et al. 2022), and a persistent contamination of the environment, feed, and animals is caused by the ubiquitous nature of *Salmonella* spp. because of their adaptation to animal and plant hosts, their ability to survive in harsh settings, and their increased capacity to form biofilms (Velhner et al. 2018).

Over time, understanding of *Salmonella* spp.'s virulence mechanisms, resistance to antibiotics, and capacity to build biofilms in the poultry sector has grown (Velhner et al. 2018). In addition to antibiotics, vaccinating chickens and adding feed supplements like prebiotics, probiotics, postbiotics, synbiotics, and bacteriophages are being used to reduce *Salmonella* in poultry. However, despite research and significant investments to improve poultry management, none of the methods used throughout the food production process completely eliminate *Salmonella* spp. (Shaji et al. 2023). In eastern China in 2019, genomic analysis revealed a diverse resistome comprising 44 distinct acquired antimicrobial resistance (AMR) genes. Notably, the study identified several dominant genetic determinants responsible for phenotypic resistance across multiple drug classes: aac(6′)‐Iaa (aminoglycosides), bla_TEM‐1B_ (β‐lactams), floR (phenicols), dfrA14 (trimethoprim), fosA7 (fosfomycin), mph(A) (macrolides), qnrS1 (quinolones), sul1 (sulfonamides), tet(A) (tetracyclines), and ARR‐3 (rifampin) (Tang et al. 2022). This situation is highlighted by the spread of similar transmissible genetic components expressing antibiotic resistance genes from poultry to people and/or MDR *Salmonella* clones (Antunes et al. 2016).

Despite the expanding body of literature on AMR in Bangladesh's poultry sector, a critical research gap persists, driven by the prevalence of small‐scale studies that select antibiotics arbitrarily and lack complete phenotypic profiling. Existing surveillance often focused on antimicrobials not frequently used in local poultry production, thereby overlooking the most prevalent and specific serovars, such as Gallinarum and Pullorum, and their corresponding AMR genes. A reliance on samples taken from busy locations, such as slaughterhouses and sales centers, rather than directly from farms, exacerbates this data fragmentation. This methodological approach does not allow a clear distinction between farm‐level resistance and secondary contamination from diverse external strains present in the market environment. This research gap poses a severe threat to evidence‐based intervention. The lack of standardized farm‐to‐fork traceability leads to inaccurate characterization of the AMR burden, potentially misguiding veterinary clinical protocols and national policy frameworks. Without addressing these “blind spots,” the silent proliferation of MDR pathogens through the food chain may continue unabated, undermining both animal welfare and the efficacy of critical human therapeutics.

The current study allowed an in‐depth investigation of the bacteria's AMR profile, including the identification of the most effective antibiotics, MDR status, development of the phenotypic pattern with commonly used antibiotics, and the distribution of resistance‐associated genes in poultry.

## 2 Materials and Methods

### 2.1 Ethical Statement

The research ethics of the Hajee Mohammad Danesh Science and Technology University (HSTU), Dinajpur‐5200, Bangladesh, were followed in conducting this study. Samples were collected following the verbal informed consent of the respective farm owners, and all postmortem examinations were conducted by experienced veterinarians to ensure clinical accuracy.

### 2.2 Research Framework and Field Site

This research focused on a specific study area in the eastern part of Bangladesh, known for its high chicken population density. The study was conducted over an extended 18‐month period, commencing in November 2023 and concluding in April 2025, ensuring a thorough and longitudinal analysis. To determine the sample size (*n*) using Cochran's formula with *p* = 5.66% (0.0566) (Shen et al. 2023), where *p* represents predicted prevalence, we made two conventional assumptions: *Z* = 1.96 for a 95% confidence interval level and a margin of error (*e*) = 0.05. Finally, a minimum sample size of 83 was selected.

$$n=\frac{Z^{2}\times p\times (1-p)}{e^{2}}.$$

**Figure 1:**
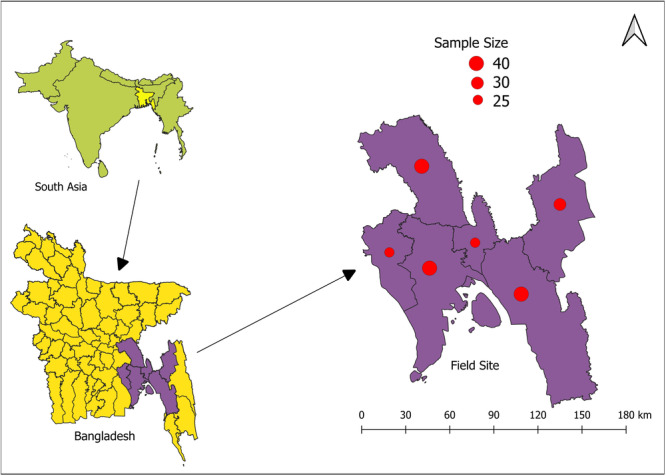
Field site map displaying the investigation area.

A total of 200 samples were collected for this study from six districts, like Cumilla (40 samples), Noakhali (40 samples), Lakshmipur (25 samples), Feni (25 samples), Chittagong (40 samples), and Khagrachori (30 samples) (Figure 1).

### 2.3 Criteria for Farm Selection and Sample Collection

The criteria for selecting farms for the study were specified. The criteria included farms that had been in operation for at least 24 months prior to the research, as well as farms with at least 2000 broiler, layer, or indigenous chickens. The sampling strategy was targeted, focusing on sick chickens exhibiting clear clinical signs of salmonellosis (Matos et al. 2021; Kumari et al. 2013). To maximize diagnostic accuracy, samples were collected from three distinct organs: cloacal swabs from live or dead birds, and intestinal and liver swabs, collected exclusively from dead chickens during postmortem examination to confirm systemic or local infection.

### 2.4 Isolation and Molecular Detection of *Salmonella enterica* Serovar Gallinarum–Pullorum from Chickens

Standard bacteriological protocols for *S. enterica* serovar Gallinarum–Pullorum isolation were meticulously followed, with all necessary media purchased from HiMedia Ltd, India, and prepared according to the manufacturer's instructions. Pre‐enrichment in buffered peptone water was the first step in the technique. To promote the quick swarming of *S. enterica* serovar Gallinarum–Pullorum, this was followed by inoculation onto Modified Semisolid Rappaport‐Vassiliadis (MSRV) medium supplemented with novobiocin. The medium was then selectively incubated at 42°C for 24 h. After streaking the presumed positive MSRV inoculum (positive reaction, colorless to light pink zone) onto xylose‐lysine‐deoxycholate agar, it was cultured at 37°C for 24 h to produce isolated colonies (red with or without black centers) that could be definitively identified.

The proteinase K/boiling method, first developed by Kapperud et al. (1993) and later improved by Estrada et al. (2007), was used to extract DNA. The resultant supernatant, which contained the extracted genomic DNA, was collected, kept at −20°C, and then used for polymerase chain reaction (PCR) to detect *S. enterica* serovar Gallinarum–Pullorum and its associated antibiotic resistance genes.

CigR was the target gene for detection, and the primers used were cigR‐F, cigR‐R1, and cigR‐R2 (Table 1). The three primers (0.4 µmol L^−1^ cigR‐F primer and 0.2 µmol L^−1^ cigR‐R1 and cigR‐R2 primers) were mixed with template DNA (1 μL) and Taq Master mix (12.5 μL of 2X master mix) in a single tube. Then, multiplex PCR amplifications began with a denaturation stage at 95°C for 3 min, followed by 30 cycles of denaturation at 95°C for 15 s, an annealing step at 50°C for 15 s, an extension at 72°C for 30 s, and a final extension at 72°C for 10 min (Zhou et al. 2020). According to Boffey (1984), the entire agarose gel electrophoresis process was carried out to obtain findings.

**Table 1:** Primer for the detection and amplification of the cigR gene in *Salmonella enterica* serovar Gallinarum–Pullorum.

Target gene	Primer	Sequence (5′–3′)	Amplicon size (bp)	Annealing temperature (°C)	Reference
cigR	cigR‐F	ATGAATAATCGTCGTGGTTT	421	50	Zhou et al. (2020)
	cigR‐R1	TAATAATCGCCGTGACCACC			
	cigR‐R2	GTAGCGCTCAGGGAAAACG			

### 2.5 Antimicrobial Susceptibility Testing (AST) Done for *S. enterica* Serovar Gallinarum–Pullorum

AST of *Salmonella* isolates was performed using the disc diffusion method, adhering strictly to the guidelines established by the Clinical and Laboratory Standards Institute (CLSI 2024). This analysis included a panel of 10 antimicrobials commonly employed for treating *S. enterica* serovar Gallinarum–Pullorum infections in Bangladesh based on the recommendation by the World Organization for Animal Health. The antimicrobial commercial discs from Oxoid, UK, and interpretive criteria for the resulting zones of inhibition for Enterobacteriaceae were based on CLSI recommendations (CLSI 2024), with isolates classified as resistant, intermediate, or susceptible (Table 2). Furthermore, an isolate was classified as MDR if it showed resistance to more than two different antimicrobial classes (Weill et al. 2006).

**Table 2:** Disc concentration and zone of inhibition interpretive criteria for *Salmonella enterica* serovar Gallinarum–Pullorum in chickens.

SL No.	Name of antibiotics with disc concentration	Sensitive zone diameter (mm)	Intermediate zone diameter (mm)	Resistance zone diameter (mm)
1	Norfloxacin (NOR‐10)	17	13–16	12
2	Enrofloxacin (ENR‐5)	23	19–22	18
3	Neomycin (N‐30)	16	14–15	13
4	Gentamicin (CN‐10)	18	15–17	14
5	Trimethoprim–sulfamethoxazole (SXT‐25)	16	11–15	10
6	Amoxicillin (AML‐10)	18	14–17	13
7	Amoxicillin + clavulanic acid (AMC‐30)	18	14–17	13
8	Cephalexin (CL‐30)	18	14–17	13
9	Tetracycline (TE‐30)	15	12–14	11
10	Florfenicol (FFC‐30)	19	15–18	14

*Source:* The Clinical and Laboratory Standards Institute (2024).

### 2.6 Detection of Antibiotic Resistance Genes for *S. enterica* Serovar Gallinarum–Pullorum

All *Salmonella* isolates were tested for the presence of the bla_CMY_ (gene for amoxicillin resistance); bla_TEM_ and bla_CTX_ (genes for amoxicillin–clavulanic acid/cephalexin resistance); sul1, sul2 and dfr A (genes for trimethoprim–sulfamethoxazole resistance); aph (gene for neomycin resistance); aac(3)‐VIa and aadB (genes for gentamicin resistance); tetA (gene for tetracycline resistance); floR (gene for florfenicol resistance); gyrA and parC (genes for quinolones resistance) genes by conventional PCR assay using the specific sets of primers as described in Table 3. The master mix, forward primer, reverse primer, nuclease‐free water, and extracted DNA were all combined in a PCR tube to create the PCR result (Table 3). A positive control reaction using a template of known size and suitable primers was constructed, as was a negative control reaction lacking template DNA. After that, the tubes were put in the thermal cycler, and the ideal PCR conditions were set.

**Table 3:** Primer sequences used in the polymerase chain reaction (PCR) to detect antimicrobial resistance genes.

SL No.	Genes for resistance	Primer name	Primer sequence (5′–3′)	Annealing temperature (°C)	Amplicon size (bp)	References
1	bla_CMY_	CMY‐F	ATGATGAAAAAATCGTTATGCT	50	1140	Daniela et al. (2014)
		CMY‐R	TTATTGCAGCTTTTCAAGAATGCG			
2	bla_TEM_	TEM‐F	ATAAAATTCTTGAAGACGAAA	53	1080	Weill et al. (2004)
		TEM‐R	GACAGTTACCAATGCTTAATC			
3	bla_CTX_	CTX‐F	CCCATGGTTAAAAAACACTGC	57	950	Schmitt et al. (2007)
		CTX‐R	CAGCGCTTTTGCCGTCTAAG			
4	sul1	sul1‐F	CGGCGTGGGCTACCTGAACG	55	433	Sunde (2005)
		sul1‐R	GCCGATCGCGTGAAGTTCCG			
5	sul2	sul2‐F	CCTGTTTCGTCCGACACAGA	60	435	Chang et al. (2007)
		sul2‐R	GAAGCGCAGCCGCAATTCAT			
6	dfr A1	D1	ACGGATCCTGGCTGTTGGTTGGAC	58	257	Lee et al. (2001)
		D2	GCAATTCACCTTCCGGCTCGATGTC			
7	aph(3′)‐IIa	aph(3′)‐IIa‐ F	CTTGAAACATGGCAAAGGTAG	55	582	Ma et al. (2007)
		aph(3′)‐IIa‐ R	AGCCGTTTCTGTAATGAAGGA			
8	aac(3)‐VIa	aac(3)‐VIa‐ F	CGCTCAGGCGATATGGTGAT	56	466	Haines (2000)
		aac(3)‐VIa‐ R	CATAATGGAGCGCGGTGACT			
9	aadB	aadB‐F	GCGAAATCTGCCGCTCTG	60	412	Ma et al. (2007)
		aadB‐R	TGCGAGCCTGTAGGACTC			
10	tetA	tetA‐F	GCTACATCCTGCTTGCCTTC	58	210	Ng et al. (2001)
		tetA‐R	CATAGATCGCCGTGAAGAGG			
11	floR	StCM‐L	CACGTTGAGCCTCTATATGG	60	888	Ahmed et al. (2007)
		StCM‐R	ATGCAGAAGTAGAACGCGAC			
12	gyrA	stgyrA1 (F)	CGTTGGTGACGTAATCGGTA	52	251	Eaves et al. (2002)
		stgyrA2 (R)	CCGTACCGTCATAGTTATCC			
13	parC	parC‐F	ATGAGCGATATGGCAGAGCGCCTTGCGCTA	60	480	Gopal et al. (2016)
		parC‐R	ACGCGCCGGTAACATTTTCGGTTCCTGCAT			

In each reaction well, we included a competitive internal amplification control (IAC). A different fluorescent probe was used to co‐amplify this control with the target DNA. We successfully eliminated false negatives caused by PCR inhibitors (such as humic acids or heme) or technical issues by ensuring the IAC was amplified in each negative sample. We used three no‐template controls (NTCs) per 96‐well plate, carefully positioned at the start, middle, and end of the loading procedure, to assess reagent purity and lab cleanliness. The entire plate was deemed compromised if any NTC displayed a *Cq* value below 40 (or the defined limit of detection).

Every sample from the original DNA extract that first produced “equivocal” results (such as *Cq* > 40) was automatically retested in duplicate. A second technician who was blind to the first results reanalyzed a random subset of 5% of all decisive positive and negative isolates to guarantee intra‐assay consistency. The primary and confirmatory tests had a 100% concordance rate.

According to Boffey (1984), the entire agarose gel electrophoresis process was carried out to obtain findings.

### 2.7 Equation of Prevalence and Multiple Antibiotic Resistance (MAR) Index

The prevalence was estimated by dividing the number of positive cases, which referred to “*Salmonella enterica* serovar Gallinarum–Pullorum found in the samples,” by the total number of tested isolates and then multiplying by 100. The MAR Index for a single isolate was calculated by dividing the number of antibiotics against which the isolate was resistant by the total number of antibiotics tested.

$$\mathrm{Prevalence}\;\left(\%\right)=\frac{\mathrm{The}\;\mathrm{number}\;\mathrm{of}\;\mathrm{positive}\;\mathrm{cases}}{\mathrm{The}\;\mathrm{total}\;\mathrm{tested}\;\mathrm{isoltes}}\times 100,$$

$$\mathrm{MAR}\;\mathrm{Index}=\frac{\mathrm{The}\;\mathrm{number}\;\mathrm{of}\;\mathrm{antibiotics}\;\mathrm{to}\;\mathrm{which}\;\mathrm{the}\;\mathrm{isolate}\;\mathrm{showed}\;\mathrm{resistance}}{\mathrm{The}\;\mathrm{total}\;\mathrm{number}\;\mathrm{of}\;\mathrm{antibiotics}\;\mathrm{to}\;\mathrm{which}\;\mathrm{the}\;\mathrm{isolate}\;\mathrm{was}\;\mathrm{tested}}.$$

### 2.8 Statistical Methods

Statistical summaries and data management were performed in Microsoft Excel Version 2024 (Microsoft Corporation 2024). Statistical precision was rigorously assessed by calculating 95% confidence intervals for all proportions, supplemented by the application of the Binomial exact test. A chi‐square goodness‐of‐fit test was used to investigate relationships between various explanatory factors. The *p* value (*p* < 0.001) was used as the level of significance. Spatial data were mapped using QGIS version 3.44.6 (QGIS Association 2026). All bar graphs and heatmaps were generated in GraphPad Prism Version 10.0.0 (GraphPad Software 2023), and the UpSet plot was generated by R Version 4.5.3 (R Core Team 2026) with the UpSetR package (Conway et al. 2017).

## 3 Results

### 3.1 Antibiotic Resistance Profile of *S. enterica* Serovar Gallinarum–Pullorum Isolated From Chickens

**Figure 2:**
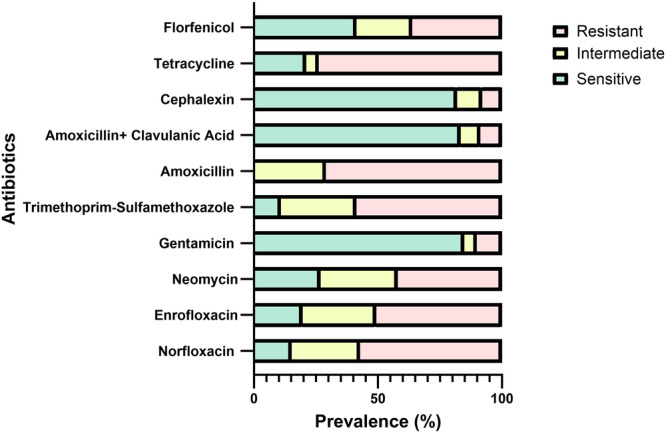
Graphical overview showing antibiotic resistance profile of *Salmonella enterica* serovar Gallinarum–Pullorum isolated from Chickens.

**Figure 3:**
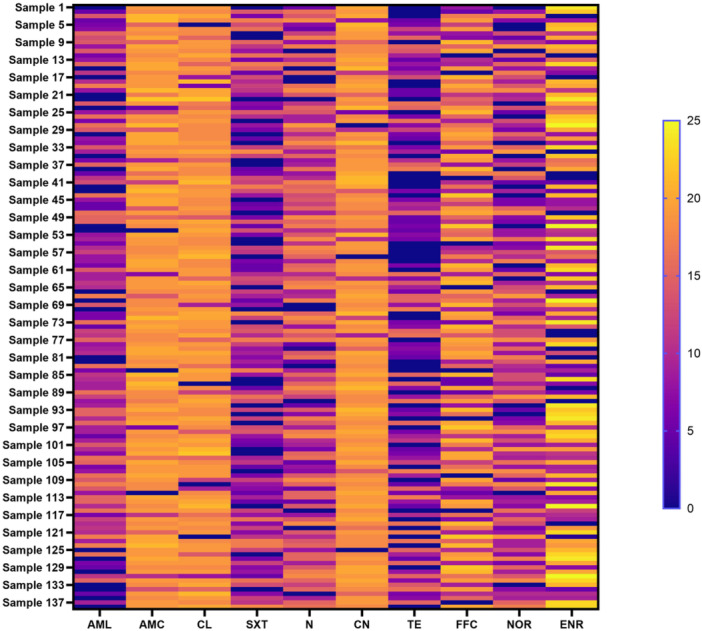
Heatmap depicting the actual antimicrobial sensitivity profile (zone of inhibition in millimeters) of the isolated samples in relation to their inhibitory activity. “No zone of inhibition (0 mm)” is shown by blue, “small zone of inhibition (~ 10 mm)” by purple, and “large zone of inhibition (≥ 25 mm)” by yellow. AMC, amoxicillin + clavulanic acid; AML, amoxicillin; CL, cephalexin; CN, gentamicin; ENR, enrofloxacin; FFC, florfenicol; N, neomycin; NOR, norfloxacin; SXT, trimethoprim–sulfamethoxazole; TE, tetracycline.

On the basis of the provided data from 138 total tested samples, there were significant differences in the effectiveness of the 10 tested antibiotics (Figure 2). Gentamicin exhibited the highest therapeutic efficacy, with 84.78% of isolates classified as sensitive, followed closely by the combination of amoxicillin–clavulanic acid at 83.33% and cephalexin at 81.88%. The highest resistance frequency was recorded for tetracycline at 73.91%, followed by amoxicillin at 71.01%. Notably, amoxicillin showed 0% sensitivity, though 28.99% of isolates fell within the intermediate category. Norfloxacin and enrofloxacin faced resistance rates of 57.24% and 50.72%, respectively. Supporting Information includes a full review of the antibiogram profile (Table S1) as well as the heatmap (Figure 3) showing the precise zone of inhibition in millimeters of each isolate.

### 3.2 Determination of MAR Index and Identification of MDR Strains of *S. enterica* Serovar Gallinarum–Pullorum

The MAR Index ranged from 0.1 to 0.7, with a highly significant correlation between the number of resistant agents and isolate frequency (*p* < 0.001). Over 99.28% of the isolates exhibited a MAR index of 0.2, indicating a high‐risk source of contamination where antibiotics were frequently used (Table 4). Correspondingly, the isolates exhibited extensive MDR profiles, with 89.13% of the population showing resistance to two or more antimicrobial agents (Table 4). The most prevalent MDR pattern involved resistance to four antimicrobial agents, observed in 31.16% of isolates (*n* = 43; 95% CI, 23.55–39.59), followed by resistance to five agents (22.46%, *n* = 31) and three agents (19.57%, *n* = 27). Notably, a subset of isolates displayed extreme resistance to 6 (10.87%) and 7 (5.07%) antimicrobial classes.

**Table 4:** Frequency of multidrug‐resistant strains of *Salmonella enterica* serovar Gallinarum–Pullorum in chickens.

SL No.	Number of resistant antimicrobials	Number of resistant isolates	Prevalence (95% confidence interval)	MAR index	Level of significance
1	1	1	0.72 (0.02–3.97)	0.1	(*p* < 0.001)***
2	2	14	10.15 (5.66–16.44)	0.2	
3	3	27	19.57 (13.31–27.18)	0.3	
4	4	43	31.16 (23.55–39.59)	0.4	
5	5	31	22.46 (15.80–30.34)	0.5	
6	6	15	10.87 (6.21–17.29)	0.6	
7	7	7	5.07 (2.06–10.17)	0.7	
**Total**		138	100		

Abbreviation: MAR, multiple antibiotic resistance.

### 3.3 MDR Pattern of *S. enterica* Serovar Gallinarum–Pullorum

**Figure 4:**
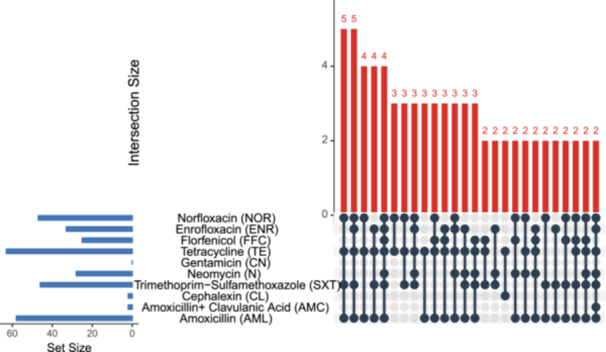
Upset plot showing phenotypic resistance pattern of *Salmonella enterica* serovar Gallinarum–Pullorum in chickens.

The MDR pattern was based on 10 antimicrobials (Figure 4). The most frequently observed patterns, each found in five isolates (3.62%; 95% CI, 1.19–8.25), are AML‐SXT‐TE‐NOR and AML‐SXT‐TE‐NOR‐ENR. Following closely were three patterns observed in four isolates each (2.90%; 95% CI, 0.8–7.25): AML‐SXT‐N‐TE‐FFC‐NOR‐ENR, AML‐TE‐NOR, and AML‐SXT‐TE‐ENR. The majority of the remaining patterns were found in small numbers, with six patterns observed in three isolates each (2.17%; 95% CI, 0.45–6.22) (including AML‐TE, SXT‐TE‐NOR, and AML‐SXT‐N‐TE‐FFC), and the largest group, 14 distinct patterns, observed in only two isolates each (1.45%; 95% CI, 0.18–5.14) (such as CL‐TE and AML‐N‐FFC‐NOR). The phenotypic resistance pattern of each isolate (*n* = 138) is provided as Supporting Information (Table S2).

### 3.4 Distribution of Antibiotic Resistance Genes in *S. enterica* Serovar Gallinarum–Pullorum

**Figure 5:**
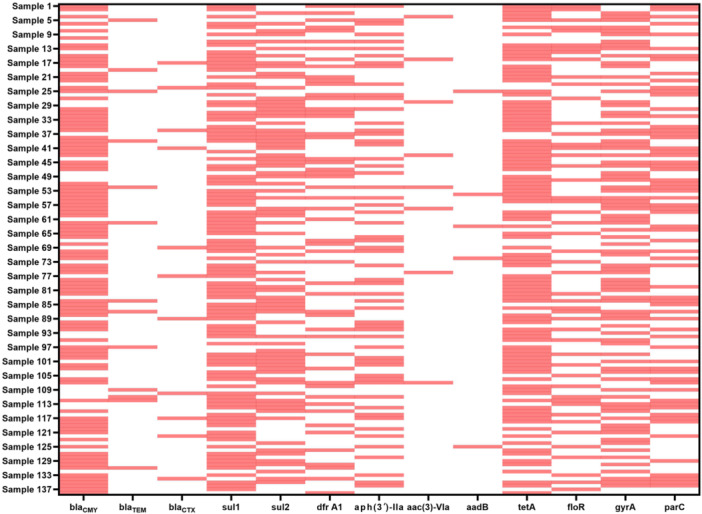
Heat map displaying molecular profiling of antibiotic resistance genes in *Salmonella enterica* serovar Gallinarum–Pullorum of 138 samples. Light pink indicates positive.

Molecular characterization of the 138 *Salmonella* isolates revealed a high frequency of resistance genes, with the occurrence of several determinants being highly significant (*p* < 0.001) (Table 5). The most prevalent gene identified was tetA (74.64%, *n* = 103; 95% CI, 66.53–81.66), followed by the sulfonamide resistance gene sul1 (73.19%, *n* = 101) and the beta‐lactamase determinant bla_CMY_ (71.01%, *n* = 98). In the quinolone class, gyrA and parC were detected in 60.87% and 47.83% of isolates, respectively. Conversely, the lowest genotypic prevalence was observed for gentamicin resistance genes, with aac(3)‐VIa and aadB present in only 5.80% and 3.62% of the population, respectively, and other beta‐lactamase determinants, bla_TEM_ and bla_CTX_, were detected in 9.42% and 7.97%, respectively. Other identified determinants included sul2 (55.80%), aph(3′)‐IIa (42.03%), dfr A1 (37.68%), and floR (35.51%). Each isolate's antibiotic resistance genes were shown in a heat map (Figure 5) and Table S3.

**Table 5:** Occurrence of antimicrobial resistance genes in *Salmonella enterica* serovar Gallinarum–Pullorum (*n* = 138) from chicken.

SL No.	Name of antibiotics	Resistance genes	Number of positive isolates	Prevalence (95% confidence interval)	Level of significance
1	Amoxicillin	bla_CMY_	98	71.01 (62.69–78.42)	(*p* < 0.001)***
2	Amoxicillin + clavulanic acid/cephalexin	bla_TEM_	13	9.42 (5.11–15.57)	
3		bla_CTX_	11	7.97 (4.05–13.81)	
4	Sulfamethoxazole	sul1	101	73.19 (64.99–80.37)	
5		sul2	77	55.80 (47.10–64.24)	
6	Trimethoprim	dfr A1	52	37.68 (29.58–46.32)	
7	Neomycin	aph(3′)‐IIa	58	42.03 (33.68–50.72)	
8	Gentamicin	aac(3)‐VIa	8	5.80 (2.54–11.10)	
9		aadB	5	3.62 (1.19–8.25)	
10	Tetracycline	tetA	103	74.64 (66.53–81.66)	
11	Florfenicol	floR	49	35.51 (27.55–44.10)	
12	Quinolones	gyrA	84	60.87 (52.20–69.06)	
13		parC	66	47.83 (39.26–56.49)	

## 4 Discussion

The emergence of antibiotic‐resistant *Salmonella* spp. in improperly handled poultry, driven by the over‐ and misuse of antibiotics, poses a grave public health crisis by potentially rendering treatments ineffective during fatal foodborne outbreaks (Punchihewage‐Don et al. 2022). Gentamicin (84.78%), amoxicillin–clavulanic acid (83.33%), and cephalexin (81.88%) demonstrated the highest levels of antimicrobial sensitivity in this study. These findings contrasted with recent geographical data; for instance, Cortés et al. (2022) reported significantly higher gentamicin resistance (57.0%) in Eastern Spain (2015–2017), while Polat et al. (2025) observed increased resistance to amoxicillin–clavulanic acid (39.5%) in broiler samples from northwest Turkey (2023–2024). Conversely, the highest resistance rates in this cohort were attributed to tetracycline (73.91%), amoxicillin (71.01%), trimethoprim–sulfamethoxazole (58.70%), and norfloxacin (57.24%). While Nguyen et al. (2021) similarly identified tetracycline (55.80%) and sulfamethoxazole–trimethoprim (53.04%) as primary resistant agents in Vietnamese poultry *Salmonella* (2017–2020), the current study highlighted a more severe resistance profile. Notably, resistance to tetracycline, norfloxacin, and beta‐lactam penicillins (lacking clavulanic acid) was markedly higher in our findings, with amoxicillin failing to show any susceptible samples. These findings were subject to regional variations in antibiotic prescription patterns, as well as discrepancies in sample size, geographical location, and specific poultry breeds and strains or bacterial strains.

According to Krumperman (1983), Values exceeding the 0.2 threshold indicate that the isolates have likely undergone selective pressure due to anthropogenic antibiotic use, facilitating the accumulation of resistance determinants. The MAR Index, which ranged from 0.2 to 0.7 in this study, indicated a higher risk of MDR. The MAR index of this study was lower than the results of Mir et al. (2022) in southeast Iran (0.45–0.81), as they tested antibiotics that might not be specific to *Salmonella* species, such as tylosin and tiamulin. Furthermore, Fagbamila et al. (2023) reported MAR indices ranging from 0.07 to 0.5 in layer chicken farms in Nigeria, which were significantly lower than those observed in the present work. This discrepancy likely originated from their selection of antibiotics, specifically cefotaxime, ceftazidime, and meropenem, which were not commonly utilized in poultry production in Bangladesh.

Shen et al. (2023) reported considerably high MDR in 43.52% of all *Salmonella* isolates in a study conducted in China from 2019 to 2022, and in another investigation in Southern Italy, Di Taranto et al. (2025) obtained 112 of 128 isolates (87.5%). On the other hand, the MDR group accounted for 123 isolates, resulting in a commanding combined prevalence of 89.13%, and four resistant drugs accounted for the highest frequency of resistance in the MDR group, with a peak prevalence of 31.16% in this research. The variability in these reports originated from the lack of strain‐specific analysis, alongside temporal and geographical discrepancies, despite a shared focus on poultry‐based research.

The AMR pattern that was observed in this investigation illustrated a significant clustering of resistance toward amoxicillin, sulfamethoxazole–trimethoprim, and tetracyclines, frequently co‐occurring with fluoroquinolone resistance. The most prevalent AMR patterns were the AML‐SXT‐TE‐NOR and AML‐SXT‐TE‐NOR‐ENR, each identified in five isolates (3.62%), and AML‐SXT‐N‐TE‐FFC‐NOR‐ENR, AML‐TE‐NOR, and AML‐SXT‐TE‐ENR were observed in four isolates each (2.90%). Dlamini et al. (2024) investigated the AMR profiles of *Salmonella* within the Ngaka Modiri Molema District of South Africa's North West Province, analyzing 75 cloacal fecal samples obtained from a stratified cohort of 15 poultry holdings comprising five layer, five broiler, and five indigenous dual‐purpose chicken farms. The study identified significant MDR trends, with the most prevalent phenotypes being SXT‐W‐TE (16%), E‐SXT‐W‐TE (13%), and the extensive AMP‐AML‐E‐SXT‐W‐TE (10%) pattern. While these findings correlated with the current study, the observed disparities in resistance patterns and percentages emphasized the influence of serovar‐specific characteristics and the varying selective pressures exerted by localized antibiotic administration protocols.

Shen et al. (2023) found significantly higher frequencies of bla_TEM_ (61.11%) and bla_CMY‐2_ (63.89%), and Zhao et al. (2023) found a tetA prevalence of 81.4% in Chinese isolates. In this investigation, the genetic landscape of AMR was dominated by high prevalences of tetA (74.64%), sul1 (73.19%), and the bla_CMY_ beta‐lactamase gene. Moderate frequencies were observed for gyrA (60.87%), sul2 (55.80%), parC (47.83%), and aph(3′)‐IIa (42.03%), indicating diverse pathways for MDR. Conversely, resistance determinants for certain beta‐lactams and aminoglycosides, such as bla_TEM_ (9.42%), bla_CTX_ (7.97%), and aadB (3.62%), were significantly less prevalent within the study population. While similarities in the presence of AMR genes were observed, the findings underscored pronounced regional heterogeneity in their distribution. This spatial variability suggested that varying degrees of antimicrobial selection pressure played a critical role in shaping the unique resistome profiles of specific geographic areas.

The findings of this investigation provide a critical framework for transitioning from conventional antimicrobial reliance toward a systemic antibiotic cycling as well as a replacement paradigm, and current surveillance of the AMR genes would meet the demands of a departure from localized interventions toward a comprehensive global prevention strategy. Ultimately, this work positions AMR mitigation as a cornerstone of global food security, ensuring that poultry, a primary protein source, remains a viable and safe commodity for an escalating global population. This study also suggests that effective AMR mitigation in Bangladesh necessitates a strong, coordinated One Health approach that bridges the gap between the human and animal health sectors, moving from discussion to decisive action. Implementing strict antibiotic stewardship programs in both veterinary and human medicine, with a focus on prohibiting the use of critical antimicrobials for livestock growth promotion and restricting their over‐the‐counter sales without a prescription, are key practical interventions.

A number of limitations should be taken into account to improve future research frameworks, even though this thorough study offered important insights by addressing the research gap in the phenotypic and genotypic characterization of AMR trends in *S. enterica* serovar Gallinarum–Pullorum. First, the sampling's geographic reach was somewhat limited; a more representative national or regional profile of resistance trends would result from broadening the study area to encompass a wider range of agroecological zones and production methods. Moreover, a crucial shortcoming was the lack of phylogenetic analysis. Whole‐genome sequencing (WGS) would enable accurate mapping of evolutionary relationships and the detection of mobile genetic elements or high‐risk clones that might cause horizontal gene transfer and genomic sequences, and isolated resistance genes should be deposited into public repositories like GenBank to support global data sharing and long‐term surveillance. To continue in‐depth research into the molecular causes of resistance and to develop targeted intervention strategies to reduce the public health hazards associated with zoonotic *Salmonella*, it was imperative to establish such a genomic baseline.

## 5 Conclusion

The empirical evidence synthesized in this study substantiates a high prevalence of AMR within *S. enterica* serovar Gallinarum–Pullorum isolated from poultry reservoirs. Critically, a significant majority of these isolates exhibited MDR phenotypes, a finding corroborated by the genomic identification of a diverse array of resistance‐conferring determinants, most notably bla_TEM_, tetA, and sul1. The practical significance of this research lies in its focus on antimicrobial agents currently utilized in clinical poultry management to control and treat salmonellosis, providing a direct reflection of contemporary selection pressures. While this study offered robust phenotypic and genotypic insights, a transition toward comprehensive WGS remained a necessary next step to fully elucidate the finer nuances of clonal evolution and plasmid‐mediated horizontal gene transfer. Ultimately, these findings underscore the urgent necessity for integrated “One Health” surveillance strategies to mitigate the risk of zoonotic transmission and preserve the efficacy of critical antimicrobial therapies.

## Author Contributions

**Md. Masuk Rahman Kingshuk:** conceptualization, methodology, software, data curation, formal analysis, validation, investigation, visualization, writing – original draft. **Shanzida Binte Alam:** conceptualization, methodology, data curation, investigation, formal analysis, writing – original draft, visualization. **Md. Shajedur Rahman:** conceptualization, methodology, data curation, supervision, formal analysis, validation, investigation, writing – review and editing, project administration, resources. **Md. Nurnoby Islam:** conceptualization, methodology, data curation, supervision, formal analysis, validation, investigation, funding acquisition, project administration, resources, writing – review and editing.

## Ethics Statement

The authors have nothing to report.

## Conflicts of Interest

None declared.

## Supporting information

Supporting File 1

Supporting File 2

Supporting File 3

## Data Availability

The authors confirm that the data supporting the findings of this study are available within the article and its Supporting Informations.
