# Supplementary material for: The Landscape of Salmonella enterica Serovar Gallinarum–Pullorum Antimicrobial Resistance in Bangladesh's Poultry Industry: A Combined Phenotypic and Molecular Study
Source: Microbiologyopen. 2026 Jun 10;15(3):e70328. doi: 10.1002/mbo3.70328 (PMC13253360; doi:10.1002/mbo3.70328)
Supplement: Supplementary file 1 — Supporting File 1 [file MBO3-15-e70328-s003.docx]

**Supplemental Table 1:** Table showing Overview of antimicrobial resistant profile of *Salmonella enterica* serovar Gallinarum-Pullorum isolated from Chickens

| SL No | Name of Antibiotics with Disc Concentration | Total tested sample, n=138 | | | | | |
| --- | --- | --- | --- | --- | --- | --- | --- |
|  |  | Sensitive | | Intermediate | | Resistant | |
|  |  | Quantity | % | Quantity | % | Quantity | % |
| 1 | Norfloxacin **(NOR-10)** | 21 | 15.22 | 38 | 27.54 | 79 | 57.24 |
| 2 | Enrofloxacin **(ENR-5)** | 27 | 19.57 | 41 | 29.71 | 70 | 50.72 |
| 3 | Neomycin **(N-30)** | 37 | 26.81 | 43 | 31.16 | 58 | 42.03 |
| 4 | Gentamicin **(CN-10)** | 117 | 84.78 | 7 | 5.07 | 14 | 10.15 |
| 5 | Trimethoprim-Sulfamethoxazole **(SXT-25)** | 15 | 10.87 | 42 | 30.43 | 81 | 58.70 |
| 6 | Amoxicillin **(AML-10)** | 0 | 0 | 40 | 28.99 | 98 | 71.01 |
| 7 | Amoxicillin+ Clavulanic Acid **(AMC-30)** | 115 | 83.33 | 11 | 7.97 | 12 | 8.70 |
| 8 | Cephalexin **(CL-30)** | 113 | 81.88 | 14 | 10.15 | 11 | 7.97 |
| 9 | Tetracycline **(TE-30)** | 29 | 21.02 | 7 | 5.07 | 102 | 73.91 |
| 10 | Florfenicol **(FFC-30)** | 57 | 41.31 | 31 | 22.46 | 50 | 36.23 |
