# Supplementary material for: The Landscape of Salmonella enterica Serovar Gallinarum–Pullorum Antimicrobial Resistance in Bangladesh's Poultry Industry: A Combined Phenotypic and Molecular Study
Source: Microbiologyopen. 2026 Jun 10;15(3):e70328. doi: 10.1002/mbo3.70328 (PMC13253360; doi:10.1002/mbo3.70328)
Supplement: Supplementary file 2 — Supporting File 2 [file MBO3-15-e70328-s002.docx]

**Supplemental Table 2:** Table showing Phenotypic resistance pattern of 138 isolates **(**NOR= Norfloxacin, ENR= Enrofloxacin, N= Neomycin, CN=Gentamicin, SXT= Trimethoprim-Sulfamethoxazole, AML= Amoxicillin, AMC= Amoxicillin+Clavulanic Acid, CL= Cephalexin, TE= Tetracycline, and FFC= Florfenicol).

| **Isolate Number** | **Phenotypic resistance pattern** |
| --- | --- |
| 1 | AML-SXT-N-TE-FFC-NOR |
| 2 | AML-TE-FFC-NOR |
| 3 | SXT-TE-ENR |
| 4 | AML-CN-TE-NOR-ENR |
| 5 | CL-N-TE-FFC-NOR |
| 6 | AML-N-NOR |
| 7 | SXT-TE-FFC-ENR |
| 8 | AML-SXT-FFC-NOR |
| 9 | N-TE-NOR-ENR |
| 10 | AML |
| 11 | SXT-N-NOR |
| 12 | AML-TE-FFC-ENR |
| 13 | AML-SXT-N-TE-FFC-NOR-ENR |
| 14 | TE-FFC |
| 15 | AML-N-TE-NOR-ENR |
| 16 | AML-SXT-CN-TE-FFC-ENR |
| 17 | AML-CL-N-ENR |
| 18 | AML-SXT-N-TE-NOR |
| 19 | CL-TE |
| 20 | AML-TE-ENR |
| 21 | AML-SXT-TE-FFC-NOR |
| 22 | AML-TE-NOR |
| 23 | SXT-N-FFC-NOR |
| 24 | AML-AMC-SXT-ENR |
| 25 | AML-AMC-CN-TE-NOR-ENR |
| 26 | SXT-N-TE-NOR |
| 27 | AML-SXT-N-FFC |
| 28 | SXT-CN-TE-NOR |
| 29 | SXT-CN-NOR |
| 30 | AML-SXT-N-TE-ENR |
| 31 | AML-SXT-TE-NOR |
| 32 | AML-SXT-TE-NOR-ENR |
| 33 | AML-TE |
| 34 | AML-N-TE-FFC-ENR |
| 35 | AML-TE-NOR |
| 36 | AML-AMC-SXT-N-TE-NOR-ENR |
| 37 | SXT-N-FFC-NOR-ENR |
| 38 | AML-SXT-NOR |
| 39 | AML-SXT-N-TE-ENR |
| 40 | AML-N-TE-FFC-ENR |
| 41 | AML-AMC-TE-FFC-NOR-ENR |
| 42 | AML-TE-NOR |
| 43 | AML-CN-TE-FFC-NOR-ENR |
| 44 | SXT-N-TE |
| 45 | AML-SXT-TE-NOR-ENR |
| 46 | AML-SXT-N-TE-FFC-NOR-ENR |
| 47 | AML-SXT-TE-ENR |
| 48 | SXT-N-ENR |
| 49 | SXT-TE-NOR |
| 50 | TE-FFC-NOR-ENR |
| 51 | AML-SXT-TE-NOR |
| 52 | AML-AMC-N-CN-TE-NOR-ENR |
| 53 | AML-SXT-TE-NOR-ENR |
| 54 | AML-SXT-CN-TE |
| 55 | AML-SXT-N-TE-FFC-NOR-ENR |
| 56 | AML-TE-FFC-NOR |
| 57 | AML-TE-FFC-ENR |
| 58 | AML-SXT-CN-TE-NOR-ENR |
| 59 | AML-SXT-N-TE-FFC-ENR |
| 60 | AML-SXT-TE-NOR |
| 61 | SXT-TE-NOR |
| 62 | AML-AMC-SXT-N-FFC-ENR |
| 63 | AML-CN-TE-NOR |
| 64 | AML-NOR-ENR |
| 65 | AML-SXT-TE-FFC |
| 66 | AML-N-TE-ENR |
| 67 | SXT-N-ENR |
| 68 | AML-SXT-NOR |
| 69 | CL-SXT-N-TE |
| 70 | AML-SXT-N-FFC-NOR-ENR |
| 71 | AML-TE-NOR |
| 72 | AML-CN-TE-ENR |
| 73 | SXT-TE-FFC |
| 74 | AML-N-TE-NOR-ENR |
| 75 | AML-ENR |
| 76 | AML-SXT-CN-FFC-ENR |
| 77 | CL-TE |
| 78 | AML-N-TE-NOR |
| 79 | AML-SXT-N-TE-ENR |
| 80 | AML-SXT-TE-NOR-ENR |
| 81 | AML-SXT-TE-ENR |
| 82 | AML-SXT-N-TE-FFC |
| 83 | SXT-N-TE-NOR-ENR |
| 84 | AMK-AMC-N-TE-FFC-NOR-ENR |
| 85 | AML-SXT-ENR |
| 86 | AML-SXT-N-TE-FFC |
| 87 | AML-CL-SXT-FFC-NOR-ENR |
| 88 | AML-SXT-TE-ENR |
| 89 | CL-TE-FFC-NOR-ENR |
| 90 | AML-N-TE |
| 91 | AML-N-ENR |
| 92 | AML-SXT-N-TE-FFC-NOR |
| 93 | TE-NOR |
| 94 | SXT-N-TE-NOR |
| 95 | AML-TE-FFC-NOR |
| 96 | SXT-NOR |
| 97 | AML-AMC-N-TE-ENR |
| 98 | AML-TE |
| 99 | AML-SXT-TE-FFC-NOR |
| 100 | AML-SXT-N-TE |
| 101 | SXT-N-TE-NOR-ENR |
| 102 | AML-SXT-N-TE-FFC |
| 103 | AML-SXT-TE-NOR-ENR |
| 104 | SXT-TE-NOR-ENR |
| 105 | N-FFC |
| 106 | AML-SXT-N-TE-NOR-ENR |
| 107 | AML-SXT-N-CN-ENR |
| 108 | SXT-TE-FFC |
| 109 | CL-TE-NOR-ENR |
| 110 | CL-SXT-TE |
| 111 | CL-SXT-N-FFC-ENR |
| 112 | AML-AMC-SXT-TE-FFC-NOR |
| 113 | SXT-FFC-NOR-ENR |
| 114 | SXT-N-TE-NOR-ENR |
| 115 | AML-SXT-TE-NOR |
| 116 | N-TE-FFC-ENR |
| 117 | AML-AMC-SXT-N-TE-NOR-ENR |
| 118 | AML-TE-FFC-ENR |
| 119 | AML-SXT-ENR |
| 120 | AML-N-TE-NOR |
| 121 | AML-SXT-FFC-NOR |
| 122 | CL-N-TE-ENR |
| 123 | AML-NOR |
| 124 | TE-NOR |
| 125 | AML-N-CN-FFC |
| 126 | SXT-TE-NOR |
| 127 | AML-SXT-TE |
| 128 | AML-N-FFC-NOR |
| 129 | AML-SXT-TE-ENR |
| 130 | AML-SXT-TE-NOR |
| 131 | AML-AMC-SXT |
| 132 | TE-NOR |
| 133 | AML-N-FFC-NOR |
| 134 | AML-AMC-SXT-NOR-ENR |
| 135 | AML-SXT-N-TE-FFC-NOR-ENR |
| 136 | AML-N-TE-NOR-ENR |
| 137 | AML-FFC |
| 138 | AML-TE |
